# Supplementary material for: Identification of photoperiod-induced specific miRNAs in the adrenal glands of Sunite sheep (Ovis aries)
Source: Front Vet Sci. 2022 Jul 22;9:888207. doi: 10.3389/fvets.2022.888207 (PMC9354845; doi:10.3389/fvets.2022.888207)
Supplement: Supplementary file 1 [file Data_Sheet_1.ZIP › Supplementary file/miRDeep2 file.zip!/miRDeep2 file/Know_Structure/SPLP42A2/oar-mir-541.pdf]

miRBase precursor	: oar-mir-541
Total read count	: 167
oar-miR-541-5p read count	: 155
oar-miR-541-3p read count	: 12
remaining reads	: 0

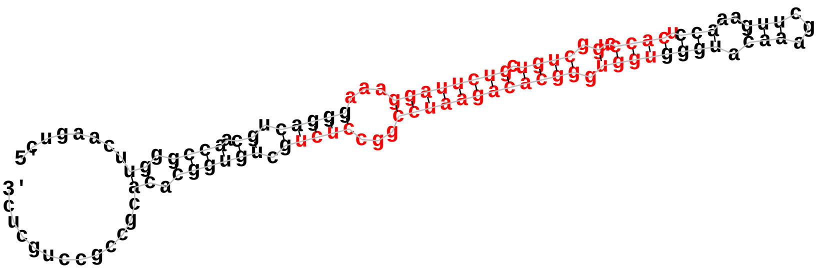

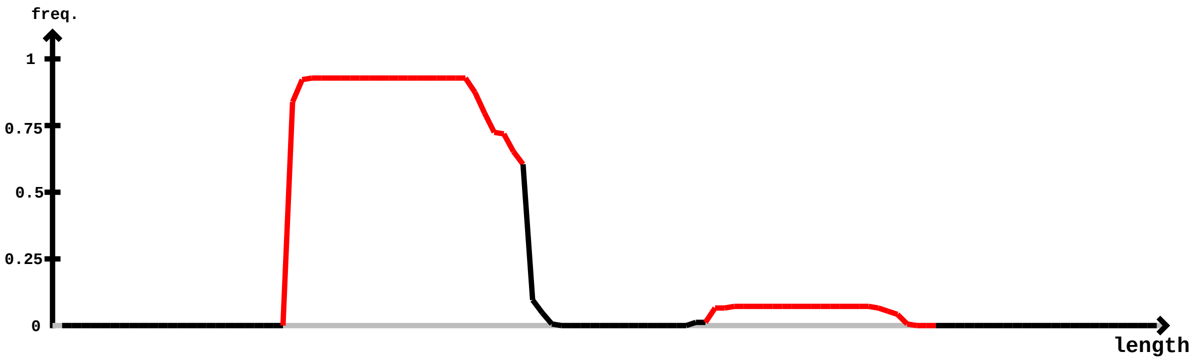


```
                                                                      oar-miR-541-3p
                           oar-miR-541-5p
5'- cugaacuugggccaacgucagggaaaggauucugcugucggucccacuccaaaguucgaaacauggguggugggcacagaauccggccucugcuguggcacacgccgccugcuc   -3'    exp
    ........((((..(((.(((((...((((((((.((((.(..((((.(((..(((...))).)))))))).))))))))))))...))))).)))(((....))))))).....   reads     mm        sample
    .......................aaaggauucugcugucggu........................................................................   9         0         seq
    .......................aaaggauucugcugucgguc.......................................................................   13        0         seq
    .......................aaaggauucugcugucggucc......................................................................   12        0         seq
    .......................aaaggauucugcugucgguccc.....................................................................   1         0         seq
    .......................aaaggauucugcugucgguccca....................................................................   11        0         seq
    .......................aaaggauucugcugucggucccac...................................................................   8         0         seq
    .......................aaaggauucugcugucggucccacu..................................................................   71        0         seq
    .......................aaaggauucugcugucggucccacuc.................................................................   8         0         seq
    .......................aaaggauucugcugucggucccacucc................................................................   7         0         seq
    ........................aaggauucugcugucggucccacu..................................................................   14        0         seq
    .........................aggauucugcugucggucccacucca...............................................................   1         0         seq
    .................................................................gguggugggcacagaauccggc...........................   2         0         seq
    ...................................................................uggugggcacagaaucc..............................   1         0         seq
    ...................................................................uggugggcacagaauccA.............................   2         1         seq
    ...................................................................uggugggcacagaauccgg............................   2         0         seq
    ...................................................................uggugggcacagaauccggc...........................   4         0         seq
    .....................................................................gugggcacagaauccggcc..........................   1         0         seq
```
